# M. Longet on the Influence of the Nerves on Muscular Irritability

**Published:** 1842-08-06

**Authors:** 


*On the influence of the Nerves on Muscular Irritability.* By M. Longet.—M. Longet found that when a nerve of voluntary motion was cut across, on the fourth day thereafter, the muscles to which it was distributed could not be excited to contract by irritating the nerve by means of a weak galvanic current. The muscles, however, supplied by this nerve contract immediately on the application of the slightest stimulus to themselves, even at the end of fifteen days. Even after the lapse of a month, direct stimulation causes them to contract slightly, and may be recognized at the end of seven weeks; no irritation of the nervous twigs, however, excites the slightest motion after the fourth day. From the seventh week, the muscular fibre, already much blanched, seems to undergo a complete degeneration, and soon ceases to contract in the slightest, even with the most powerful stimulants. It is only then in consequence of a lesion of its nutrition that muscular fibre, in losing its organic characters, loses also its essential characteristic, irritability, which, however, as has been seen, remains a long time after all nervous influence has been suppressed.—*Ibid, from Comptes Rendus des Seances de l'Academie des Sciences,* July, 1841.

